# Correlation between clonorchiasis incidences and climatic factors in Guangzhou, China

**DOI:** 10.1186/1756-3305-7-29

**Published:** 2014-01-15

**Authors:** Tiegang Li, Zhicong Yang, Ming Wang

**Affiliations:** 1 Guangzhou Center for Disease Control and Prevention, Guangdong Province 510440, China

**Keywords:** Clonorchiasis, Meteorological variables, Risk factor, Correlation, Early warning

## Abstract

**Background:**

Human infection with *Clonorchis sinensis* is still a big public health problem in Guangzhou. To investigate the correlation between clonorchiasis and climatic factors, we analyzed the clonorchiasis reported cases and simultaneous meteorological data during 2006–2012 in Guangzhou City, China.

**Findings:**

Annual incidence rate of clonorchiasis from 2006 to 2012 was 166.76, 191.55, 247.37, 213.82, 246.03, 274.71, and 239.63 (per 100 000), respectively. Each 1°C rise of temperature corresponded to an increase of 1.18% (95% CI 0.88% to 1.48%) in the monthly number of cases, and a one millimeter rise of rainfall corresponded to increase of 0.03% (95% CI 0.01% to 0.04%). Whereas each one percent rise of relative humidity corresponded to a decrease in the number of cases by 1.51% (95% CI -1.75% to -1.27%).

**Conclusions:**

We reported incidence rates of clonorchiasis showed an increasing trend by years. Temperature and rainfall were positively associated with clonorchiasis incidence, while relative humidity was inversely associated with clonorchiasis incidence. Our study provided evidence that climatic factors affect the occurrence of clonorchiasis in Guangzhou city, China.

## Findings

### Background

Clonorchiasis, also known as liver fluke disease, is a major foodborne parasitoses and caused by *Clonorchis sinensis* that parasitizes the human intrahepatic bile duct [1]. Globally, clonorchiasis is mainly distributed in East Asia and Southeast Asia, including China, Korea, Vietnam, and the Philippines [2]. It is estimated that more than 200 million people are at risk of infection, 15–20 million people are infected, and 1.5–2 million show symptoms or complications [3]. Based on the second national survey on parasitic diseases between 2001 and 2004 in China, the overall *Clonorchiasis sinensis* infection rate of the surveyed population was 0.58% [4], and Guangdong was the most endemic province with estimated infection rate of 5.36% [5].

Since 1995 Guangzhou government (capital city of Guangdong providence) has legislated to include clonorchiasis into a local reportable disease inventory [6]. This means that physicians who diagnose suspected or confirmed clonorchiasis cases must report these cases to Guangzhou Centers for Disease Control and Prevention (GZCDC) through the National Notifiable Disease Report System (NNDRS). Despite complementary interventions such as information, education and communication on safe food practices and others, which were implemented and improved by the health department, the incidence of clonorchiasis still showed a rapid increasing trend in Guangzhou [3]. Of particular note, in 2012, a total of 3,075 cases were reported, which is nearly two times the number reported in 2006 (1,662 cases). Controlling the spread of clonorchiasis infection is becoming a matter of urgency in Guangzhou [7], public health authorities are concerned about its increased incidence.

In recent decades, weather variables have been widely studied for their potential as early warning tools to fend off climate-sensitive infectious diseases such as dengue fever [8], malaria [9], and respiratory tract infections [10]. However, very little information is available for using meteorological variables to predict clonorchiasis infection. In this study, we used ecological methodology to investigate clonorchiasis epidemiological features in the subtropical city of Guangzhou for the period of 2006–2012, and compared those with the climate factors, in an effort to assess the relationship between meteorological variables and clonorchiasis, and to assist public health prevention and control measures.

## Methods

Clonorchiasis cases were obtained from NNDRS. In China, all cases of clonorchiasis were diagnosed according to the unified diagnostic criteria issued by Chinese Ministry of Health (MOH). The confirmation of diagnosis relies on 1) detecting clonorchis eggs in stool samples (Triple Kato-Katz thick smears), and/or 2) detecting worm-specific antibodies in serum samples or worm-specific antigens in serum or stool samples (ELISA or real-time PCR). Simultaneous meteorological data, including daily average temperature (in degrees Centigrade), relative humidity (as a percentage), atmospheric pressure (in hPa), wind velocity (in meters per second), sunshine (in hours) and rainfall (in millimeter) were obtained from the documentation of the Guangzhou Meteorological Bureau (GZMB). The weather data were measured at a fixed-site station located in the center district of Guangzhou. Meteorological instruments included barometers, pressure readings, thermometers, anemometers, actinometers, psychrometers, evaporimeters, and weather vanes, etc. The measurements of temperature, relative humidity, atmospheric pressure and wind velocity were usually taken every three hours before the daily average being calculated. However, for the sunshine and rainfall, the daily total was used.

Descriptive statistics (e.g. rate, proportion, mean and median) were used to describe the basic features of clonorchiasis confirmed cases in the study. A negative binomial multivariable regression was used to explore the relationship between meteorological variables and clonorchiasis. Negative binomial distribution is a Poisson distribution with an extra-dispersion term, the extra dispersion term acts as a random effect that subjects the Poisson means to additional variation that has a gamma distribution. Given the data were over-dispersed, we chose negative binomial distribution model rather than Poison model. The cases were clonorchiasis occurrence. Data were presented as the prevalence of clonorchiasis per 100,000 inhabitants grouped by month of onset. The meteorological variables were calculated by monthly average or aggregate. A preliminary analysis was conducted through Pearson’s correlation coefficient (‘r’) matrix within meteorological variables. Two separate negative binomial regression models were carried out: the first included average temperatures but no atmospheric pressure, while the second considered atmospheric pressure but no temperature. Both models included additionally relative humidity, wind velocity, sunshine, rainfall, and year as independent variables. In the final model, those variables with a P value of <0.05 in the preliminary model were included. To quantify the effects of meteorological variables, we computed the influences (e^β^ - 1), which correspond to the percent increase. The residual was checked using Pearson goodness of fit. The analyses were carried out with SAS (V.8.01, SAS Institute, Cary, New Jersey, USA).

## Results

**Figure 1:**
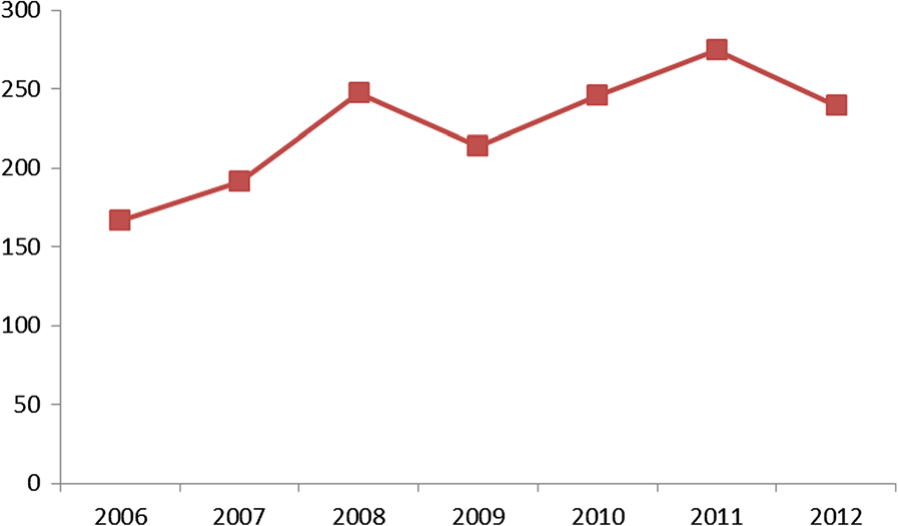
Annual occurrence of clonorchiasis confirmed cases per 100,000 in Guangzhou, China, 2006–2012.

From January 1, 2006 to December 31, 2012, a total of 18,681 clonorchiasis confirmed cases were reported in Guangzhou, of which 81.82% (15,169) were male patients and 18.80% (3,512) were female patients. Annual incidence rate from 2006 to 2012 was 166.76, 191.55, 247.37, 213.82, 246.03, 274.71, and 239.63 (per 100,000), respectively, showing an increasing trend (Figure 1). The age ranged from 0.5 to 104 years (mean age was 51.31 years). The proportion of cases between <5, 6–19, 20–44, 45–64, and >65 was 0.20% (38), 1.27% (238), 33.92% (6337), 43.11% (8,053), and 21.49% (4,015), respectively. Clonorchiasis cases were detected throughout year. The majority of cases lived in rural area (75.90%). By occupation, the highest proportion of cases was farmer, which accounted for 42.71% (7,978) of all cases, followed by housewife/househusband and retiree, which accounted for 15.21% (2,841) and 11.80% (2,204), respectively.

Summary statistics for monthly weather conditions were shown in Table 1. The correlations between independent variables revealed a strong correlation (r = -0.86, *p* < 0.01) between average temperature and atmospheric pressure (Table 2). Therefore, to avoid collinearity problems, we decided to explore the relationship of temperature and atmospheric pressure with clonorchiasis cases by using two different models, including either temperature or atmospheric pressure together with all other predictors. Of those six meteorological variables studied, temperature, relative humidity, and rainfall were statistically significant predictors of clonorchiasis cases in the final model (all *p* < 0.01). After adjusting by “year”, each 1°C rise of temperature corresponded to an increase of 1.18% (95% CI 0.88% to 1.48%) in the monthly number of clonorchiasis cases, and a one millimeter rise of rainfall corresponded to an increase of 0.03% (95% CI 0.01% to 0.04%) in monthly cases. Whereas each one percent rise of relative humidity corresponded to a decrease in the monthly number of cases by 1.51% (95% CI -1.75% to -1.27%), playing a negative effect (Table 3). Pearson goodness of fit for final model indicated *p* > 0.05.

**Table 1:** Summary statistics for monthly clonorchiasis confirmed cases and weather conditions in Guangzhou, China, 2006-2012

	**Mean**	**Std.**	**Min**	**P (25)**	**Median**	**P (75)**	**Max**
Average temperature (°C)	22.49	5.72	9.52	17.89	23.19	27.90	30.77
Average atmospheric pressure (hPa)	1007.29	6.18	994.28	1002.40	1007.83	1012.12	1018.90
Average relative humidity (%)	73.20	7.66	55.10	68.21	74.53	78.72	87.33
Average wind velocity (m/s)	1.76	0.60	1.12	1.37	1.51	2.03	3.96
Aggregate rainfall (mm)	156.10	156.64	0.10	43.77	101.60	222.62	834.72
Aggregate sunshine (h)	130.15	55.21	24.40	82.85	132.85	179.05	245.00
Clonorchiasis confirmed cases	222.39	64.01	83.00	167.50	228.00	261.50	391.00

**Table 2:** Pearson’s correlation coefficient (‘r’) matrix of meteorological variables in Guangzhou, China, 2006-2012

	**Atmospheric pressure**	**Relative humidity**	**Average temperature**	**Rainfall**	**Sunshine**	**Wind velocity**
Atmospheric pressure	1.00					
Relative humidity	-0.59 (*p* < 0.001)	1.00				
Average temp.	-0.86 (*p* < 0.001)	0.32 (*p* < 0.001)	1.00			
Rainfall	-0.58 (*p* < 0.001)	0.52 (*p* < 0.001)	0.53 (*p* < 0.001)	1.00		
Sunshine	-0.28 (*p* = 0.01)	-0.43 (*p* < 0.001)	0.40 (*p* < 0.001)	-0.16 (*p* = 0.15)	1.00	
Wind velocity	-0.10 (*p* = 0.36)	0.20 (*p* = 0.06)	-0.28 (*p* = 0.01)	-0.12 (*p* = 0.29)	0.06 (*p* = 0.99)	1.00

**Table 3:** Negative binomial regression model of meteorological factors associated with risk of clonorchiasis incidence*

	***B***	***S.E.***	***P***	**(e**^***β***^ **- 1) = percent increase (%)**	**95% CI for percent increase (%)**	
					**Lower boundary**	**Upper boundary**
**(A)**						
(Intercept)	-278.46	12.18	<0.001	-	-	-
Average temperature	0.01	0.00	<0.001	1.37	0.86	1.87
Average relative humidity	-0.02	0.00	<0.001	-1.69	-2.04	-1.34
Average wind velocity	-0.03	0.02	0.05	-3.19	-6.29	0.01
Aggregate rainfall	0.00	0.00	<0.001	0.02	0.01	0.04
Aggregate sunshine	0.00	0.00	0.13	-0.04	-0.09	0.01
Year	0.14	0.01	<0.001	15.23	13.86	16.62
**(B)**						
(Intercept)	-257.01	11.62	<0.001	-	-	-
Average atmospheric pressure	0.00	0.00	0.06	-0.48	-0.97	0.02
Average relative humidity	-0.01	0.00	<0.001	-1.37	-1.77	-0.97
Average wind velocity	-0.06	0.02	0.06	-6.03	-8.91	0.02
Aggregate rainfall	0.00	0.00	<0.001	0.03	0.02	0.05
Aggregate sunshine	0.00	0.00	0.22	0.03	-0.02	0.08
Year	0.13	0.01	<0.001	14.28	12.97	15.61
**(C)**						
(Intercept)	-255.90	8.69	<0.001	-	-	-
Average temperature	0.01	0.00	<0.001	1.18	0.88	1.48
Average relative humidity	-0.02	0.00	<0.001	-1.51	-1.75	-1.27
Aggregate rainfall	0.00	0.00	<0.001	0.03	0.01	0.04
Year	0.13	0.00	<0.001	13.94	12.97	14.91

***Note.*** *Negative binomial regression model for monthly clonorchiasis incidence without atmospheric pressure (A) and without average temperature (B). Final models (C).

CI, Confidence Interval.

## Discussion

We found the incidence of clonorchiasis showed an increasing trend by years in Guangzhou, this is consistent with the finding from northern cities of China [11]. Numerous studies revealed that occurrence of cholelithiasis is related to the unhealthy habits of residents who like to have raw fish or half-raw fish [12]. However, our study also demonstrated that more cases were observed in males, farmers, ages 45–64 years and rural residents. These findings indicated that the infection of clonorchiasis may be associated with special labor activities. In Guangzhou, it is very common for people to use human feces as fertilizer, this cultivation and breeding practice may increase the risk of clonorchiasis infection because the feces may be highly saturated with *Clonorchis sinensis* eggs [13]. Furthermore, there is evidence that human beings can become infected via the accidental ingestion of *Clonorchis sinensis* metacercariae via their hands, contaminated as a consequence of not washing after catching freshwater fish [14]. Therefore, integrated strategies and measures should be implemented to control clonorchiasis in these endemic areas.

The increasing evidence for rapid global climate change has highlighted the need for investigations examining the relationship between weather variability and infectious diseases. However, the impact of weather fluctuations on clonorchiasis is still not well understood [15]. Our current study, which was conducted in Guangzhou, demonstrated that climate factors had a significant influence on clonorchiasis infection. We found high temperature presented higher risk of clonorchiasis infection. This finding is in agreement with Liang’s findings [16], which showed that when temperature was elevated from 24.3 to 37.2 degrees C, the infection rate of clonorchiasis in the snails *Parafossarulus striatulus* and *Alocinma longicornis* increased from 12.5% to 18.0%. A possible explanation for this may be due to the fact that temperatures are important causes of variability in egg hatching [17], as well as the activities of intermediate host [18]. For example, a natural field investigation of Sichuan providence, China [19], indicated that rate of red bean snails infection with *Clonorchis sinensis* miracidia was noticeable high in the warm season (May-October) whereas almost zero in the cold season (November-March). The high temperature was believed to contribute significantly to high activities of snails.

**Figure 2:**
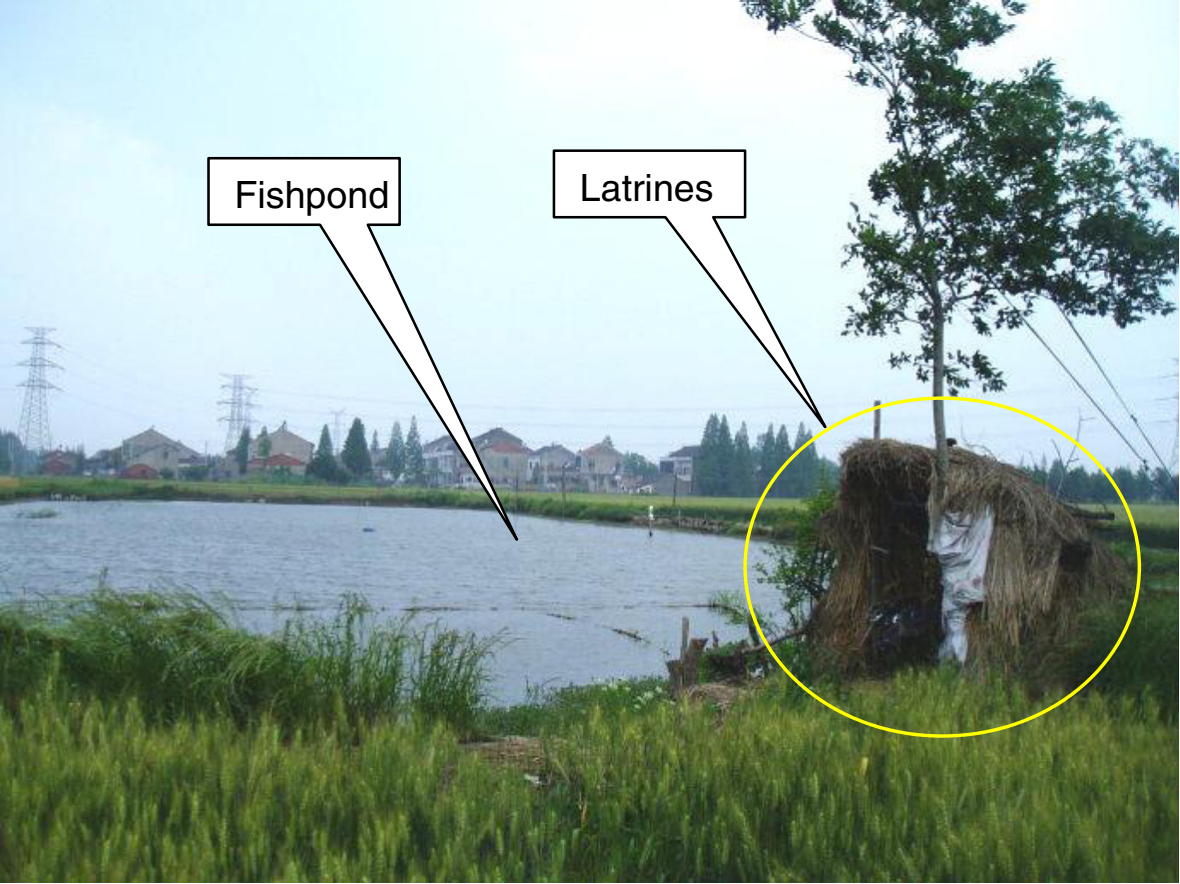
The position relationship between latrines and fishpond in Guangzhou, China.

We found rainfall positively associated with the clonorchiasis incidence. This is consistent with the findings from Sichuan province of China, which suggested that mean rainfall should be considered as the critical natural predictor for the prevalence of clonorchiasis [13]. Similarly, some published literature also indicated that rainfall were associated with schistosomiasis japonica infection in China [20,21], of which Xu XJ and his research team [22] proved that prevalence of schistosomiasis showed a significant linear regression relationship with annual rainfall. In Guangzhou, it is very common for rural households having a pond where fish are bred as a source of food. Human and animal faeces are invariably added to the ponds for nutrient enrichment. The widespread use of latrines constructed on stilts directly over fishponds or beside them is an important source of contamination (Figure 2). After rain, the faeces contain clonorchiasis eggs were washed into the ponds through natural surface drainage, which may increase the risk of susceptible snails and fish, and eventually result in human infection, especially if people persist with their traditional habits of eating raw, pickled or undercooked the fish from the pond [23].

To the best of our knowledge, the relationship between relative humidity and clonorchiasis has not been reported [13]. The result of current study showed that the relative humidity of air was negatively correlated with incidence of clonorchiasis. No literatures have been published yet revealing the underlying mechanism. A very similar report was also documented in Thailand which indicated that of 6,000 examinees, the largest number of human infections with *Opisthorchis viverrini* occurred in dry season [24]. However, when viewing the observation on schistosomes, most published studies reached the opposite conclusion. For example, a study of Pakistan reported that Pearson’s correlation between snails’ infection with schistosome cercariae and relative humidity was significantly (*P* < 0.05) positive [25]. Although the authors did not give an explanation for this, it is true that egg hatching of schistosomes needs a moist environment [26]. Therefore, the finding of the current study requires replication, especially in different areas with different weather patterns. More efforts, which focus on the mechanism of climate factors affecting the parasite infection, also need to be undertaken in future.

Some limitations must be acknowledged. First of all, we used the clonorchiasis data from NNDRS, which do not capture all cases in the community. This under-reporting of infectious can occur anywhere in the report chain, from the initial decision of patient to not seek health care to failure to record a case in the disease registry, due to the mildness or lack of symptoms. Secondly, the incubation period of 30 days for every case cannot be determined exactly. We thus chose to use monthly aggregated data of clonorchiasis and monthly average or aggregate meteorological data, the direction of these approximations are likely to be random, suggesting that our risk estimates are reliable. Thirdly, owing to this investigation being an ecological study, although we emphasized the impact of climate, we could not exclude potential confounding factors. For example, the diagnosis bias, our study did not identify clonorchiasis co-infection with intestinal flukes. It is reported that the co-infection with clonorchiasis and minute intestinal flukes (MIF) is very common in some area of China [27]. On the other hand, considering the increase of clonorchiasis reports over the years, we added the “year” as a variable in the model to control the yearly variance. Even though, reporting bias may also exist due to the data we used from NNDRS. In addition, other factors such as socio-economic factors, sanitation service levels, and personal hygiene awareness etc., need to be addressed in further studies.

## Conclusions

In conclusion, despite these limitations, we reported that the incidence of clonorchiasis showed an increasing trend by years in Guangzhou. A rise of temperature and rainfall may increase the risk of clonorchiasis infection, whereas an increase in relative humidity may reduce the risk of clonorchiasis infection. Our study provided evidence that climatic factors affect the occurrence and transmission of clonorchiasis, which may be useful for developing an early warning system.

## Competing interests

The authors declare no financial, academic or intellectual competing interests.

## Authors’ contributions

Conceived and designed the study: ZY, MW. Analyzed the data: TL. Contributed materials/analysis tools: ZY, MW. Wrote the paper: TL, ZY, MW. All authors contributed to and approved the final version of the manuscript.

## Authors’ information

The authors are all epidemiologists in Guangzhou center for disease control and prevention (GZCDC). The authors regularly conduct the surveillance on infectious disease, filed investigation on outbreak, emergent management on public health crisis, and research on risk factor and transmission of diseases.
